# Correction to “Brain‐wide versus genome‐wide vulnerability biomarkers for severe mental illnesses”

**DOI:** 10.1002/hbm.26145

**Published:** 2022-11-23

Kochunov, P., Ma, Y., Hatch, K. S., Gao, S., Jahanshad, N., Thompson, P. M., Adhikari, B. M., Bruce, H., Van der vaart, A., Goldwaser, E. L., Sotiras, A., Kvarta, M. D., Ma, T., Chen, S., Nichols, T. E., & Hong, L. E. (2022). Brain‐wide versus genome‐wide vulnerability biomarkers for severe mental illnesses. *Human Brain Mapping, 43*(16), 4970–4983. https://doi.org/10.1002/hbm.26056

In the originally published version of the article, Si Gao was inadvertently omitted as a co‐author. The article has since been corrected and the correct authorship appears below:

 

Peter Kochunov^1^ | Yizhou Ma^1^ | Kathryn S. Hatch^1^ | Si Gao^1^ | Neda Jahanshad^2^ | Paul M. Thompson^2^ | Bhim M. Adhikari^1^ | Heather Bruce^1^ | Andrew Van der vaart^1^ | Eric L. Goldwaser^1^ | Aris Sotiras^3^ | Mark D. Kvarta^1^ | Tianzhou Ma^4^ | Shuo Chen^1^ | Thomas E. Nichols^5^ | L. Elliot Hong^1^

 

^1^Maryland Psychiatric Research Center, Department of Psychiatry, University of Maryland School of Medicine, Baltimore, Maryland, USA.

^2^Imaging Genetics Center, Stevens Neuroimaging & Informatics Institute, Keck School of Medicine of USC, Los Angeles, California, USA.

^3^Institute of Informatics, University of Washington, School of Medicine, St. Louis, Missouri, USA.

^4^Department of Epidemiology and Biostatistics, University of Maryland, College Park, Maryland, USA.

^5^Nuffield Department of Population Health, University of Oxford, Oxford, UK.

